# Occupational health and health care in Russia and Russian Arctic: 1980–2010

**DOI:** 10.3402/ijch.v72i0.20456

**Published:** 2013-03-19

**Authors:** Alexey A. Dudarev, Jon Øyvind Odland

**Affiliations:** 1 Hygiene Department, Northwest Public Health Research Centre, St. Petersburg, Russia; 2 AMAP Secretariat, University of Tromsø, Tromsø, Norway

**Keywords:** occupational diseases, occupational health care, occupational safety, labour conditions, Russian Arctic

## Abstract

**Background:**

There is a paradox in Russia and its Arctic regions which reports extremely low rates of occupational diseases (ODs), far below those of other socially and economically advanced circumpolar countries. Yet, there is widespread disregard for occupational health regulations and neglect of basic occupational health services across many industrial enterprises.

**Study design and methods:**

This review article presents official statistics and summarises the results of a search of peer-reviewed scientific literature published in Russia on ODs and occupational health care in Russia and the Russian Arctic, within the period 1980–2010.

**Results:**

Worsening of the economic situation, layoff of workers, threat of unemployment and increased work load happened during the “wild market” industrial restructuring in 1990–2000, when the health and safety of workers were of little concern. Russian employers are not legally held accountable for neglecting safety rules and for underreporting of ODs. Almost 80% of all Russian industrial enterprises are considered dangerous or hazardous for health. Hygienic control of working conditions was minimised or excluded in the majority of enterprises, and the health status of workers remains largely unknown. There is direct evidence of general degradation of the occupational health care system in Russia. The real levels of ODs in Russia are estimated to be at least 10–100 times higher than reported by official statistics. The low official rates are the result of deliberate hiding of ODs, lack of coverage of working personnel by properly conducted medical examinations, incompetent management and the poor quality of staff, facilities and equipment.

**Conclusions:**

Reform of the Russian occupational health care system is urgently needed, including the passing of strong occupational health legislation and their enforcement, the maintenance of credible health monitoring and effective health services for workers, improved training of occupational health personnel, protection of sanitary-hygienic laboratories in industrial enterprises, and support for research assessing occupational risk and the effectiveness of interventions.

In the Russian Federation, “Regions of the Far North and equivalent areas” is defined by legislation as consisting of 16 “subjects” (administrative regions, such as republics, krays, oblasts and okrugs) and parts of the territories of an additional 11 subjects. Together, they occupy about 11 million km^2^ (about two-thirds of the area of Russia) where 10.7 million people live, accounting for only 7.5% of the population of the Russian Federation.

The Russian North is economically important, producing 18% of electric power, 25% of lumber, 90% of natural gas, 75% of oil, 80% of gold and diamonds and 60% of metals of the country. Hundreds of thousands of people work in these industries, most of them permanently.

Unfortunately, state authorities do not pay proper attention to the problems of people working in the North. During the last 20 years, several governmental decrees were issued, aimed at sustainable development of the Arctic territories, but these were largely ignored and not implemented. The disintegration of the Soviet Union and subsequent reforms in Russia directed at the creation of a market economy resulted in substantial changes in migration patterns in the North. Considerable out-migration was observed. Social guarantees earlier established by the state, such as income supplements, not only lost their role as an incentive to work in the North but also failed to provide an adequate standard of living. The number of residents of the Far North decreased from 12.9 million in 1990 to 10.7 million in 2006, among whom 6.2 million were classified as economically active, 1 million as unemployed, and 3.5 million as disabled. Today, the demographic situation in the North is integrally connected with that in place in Russia as a whole, characterised by a decrease in the birth rate, substantial increase in the death rate and reduction of average life expectancy at birth (1).

Between 1990 and 2000, much of the infrastructure created over decades has been substantially abandoned or deteriorated, and now across the Russian Arctic there are empty settlements, mines, factories, airports, fur farms, and so on. The present status of the Arctic regions is deplorable. Once shining lights, such as Amderma, Dickson, Tiksi and Pevek, are nowadays only tenuously connected to the outside world by irregular transport. There are some exceptions, for example, Naryan-Mar in the Nenets, Autonomous Okrug in north-western Siberia and Anadyr in Chukotka. Much of the new resource developments in oil and gas took place without regard to proper environmental protection.

## Recent governmental statements on occupational health

The deplorable state of occupational health in the country was recognised by the Russian government and was highlighted in the National Report for 2005 (2).

Annually, in the Russian Federation, 190,000 workers employed in dangerous and hazardous industries die; 15,000 workers die from occupational injuries; and 180,000 workers retire early, many for health reasons. More than 30% of deaths nationally occur among people at working-age, 4.5 times higher than in other European countries. These figures were presented by Dmitry Medvedev, the President of the Russian Federation, in 2008, during a meeting with representatives of a Russian business organisation devoted to the problems of population and health care. The President told the assembled participants: “Problems threatening the safety of people still exist in Russia; they also exist in workplaces; high mortality is not only a medical but also social problem. It is necessary to improve the health care system, particularly the preventive services.”

The Concept of the Federal Program of Actions on Improvement of Labour Conditions and Safety for 2008–2010 (3) was published in 2008, which became a part of the Concept of Demographic Policy of the Russian Federation for the period until 2025 (4). Its assessment states, in summary: “In the Russian Federation annually about 180 thousand persons die of causes related to harmful and dangerous industrial practices, about 200 thousand persons are injured occupationally, more than 10 thousand cases of occupational diseases (ODs) are registered and more than 14 thousand persons become invalid because of occupational injuries and diseases” (3).

Nikolaj Izmerov, Director of the Research Institute of Labour Medicine, and Academician of the Russian Academy of Medical Sciences, provided more gloomy data specific to women workers: “In 2007 generally in Russia 66.8 million people were employed, 33.9 million of them women, of whom 20 million were of child-bearing age. About 4 million women (12%) worked in conditions which violated sanitary-hygienic regulations and promoted ODs. According to the data of periodical medical examinations, every second working woman was chronically ill. Among women who worked in conditions where they were exposed to heat or chemicals, every sixth woman suffered from infertility, and every seventh experienced spontaneous abortions” (5).

## Official statistics on occupational health: 1980–2010

**Fig. 1:**
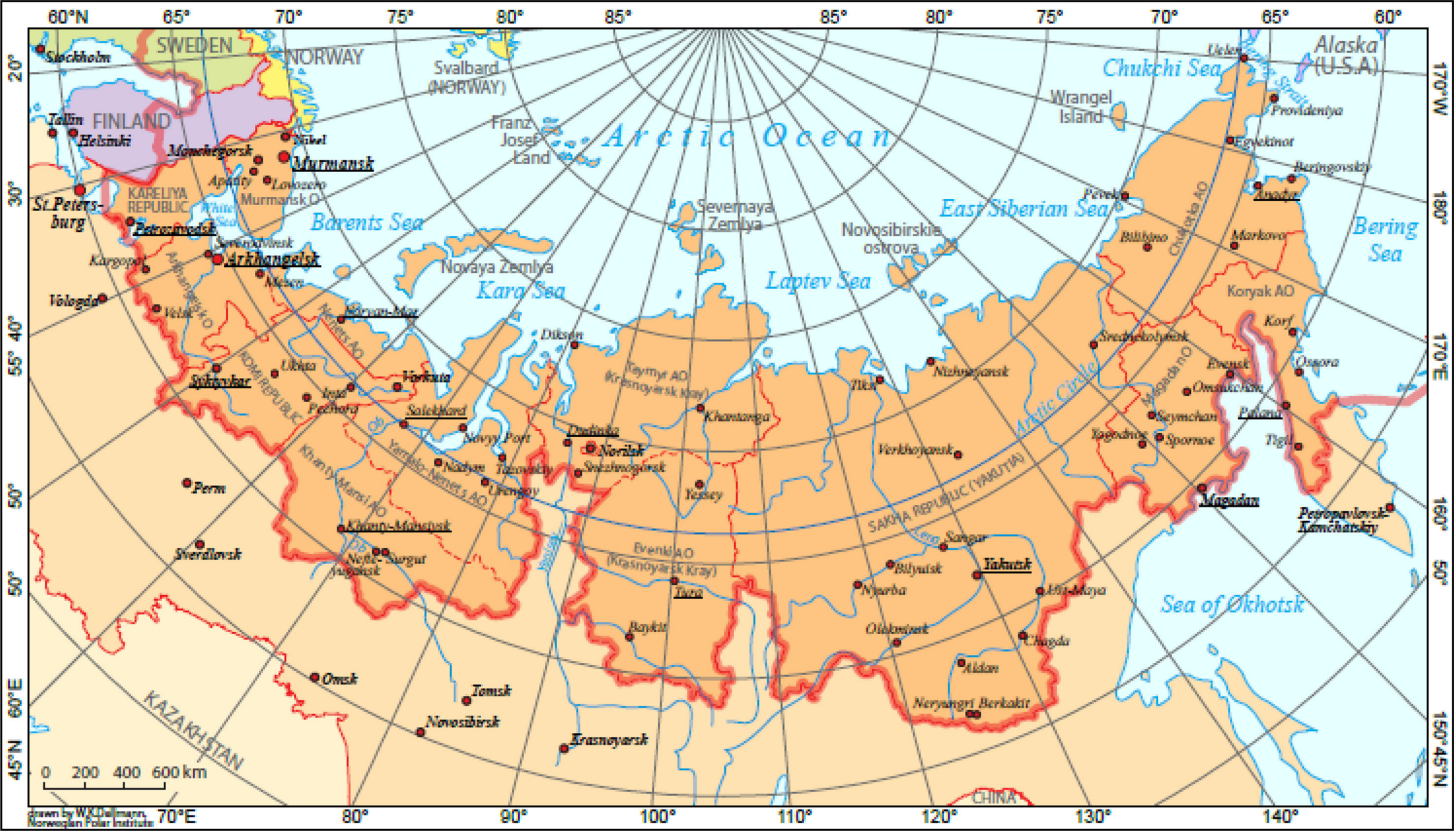
Map of northern regions of the Russian Federation. (Reproduced by permission from Young TK, ed. *Circumpolar Health Atlas*. Toronto: University of Toronto Press, 2012)

Official statistical data on ODs were available for the USSR/Russia for the period 1980–2010 from the Russian Statistical Yearbooks (6–8) and for 7 northern and far eastern regions from regional statistical yearbooks of Arkhangelsk Oblast, Murmansk Oblast, the Republic of Karelia, the Republic of Komi, Chukotka Autonomous Okrug, Kamchatka Oblast and Magadan Oblast (9–15) (Fig. 1). Regional data are available mainly for the period 2000–2008. A more complete regional picture is not possible as not all regions produced their own statistical yearbooks, or they are difficult to locate. The 7 regions (4 far-western and 3 far-eastern) nevertheless demonstrate the scope of the problem during the first decade of the century.

**Fig. 2:**
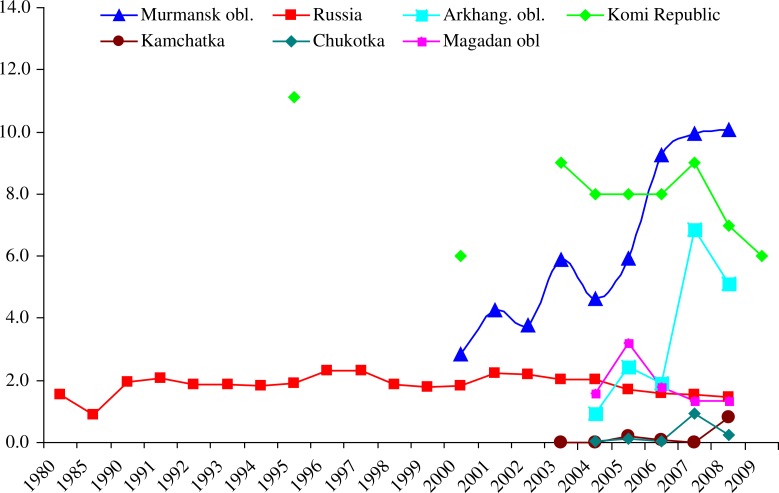
Rate of new cases of occupational diseases, including poisonings (per 10,000 workers), in Russia and selected Arctic regions.

The rate of new cases of ODs in 6 northern regions – Arkhangelsk Oblast, Murmansk Oblast, the Republic of Komi, Chukotka Autonomous Okrug, Kamchatka Oblast and Magadan Oblast compared to the whole Russia are presented in Fig. 2.

Figure 2 shows that in Russia the rate of ODs have been stable over the 30-year period, averaging 2 cases per 10,000 workers per year, which is quite low. Levels in Murmansk and Komi are 2–5 times higher than Russian rates (up to 10 cases/10,000), with Murmansk showing a steep rise and Komi a fall in rates. The situation in Arkhangelsk and Magadan are similar to that of Russia, but by mid-decade, the Arkhangelsk rate curves sharply upwards. The very low rates reported from the 2 far-eastern regions (Chukotka and Kamchatka), with almost no cases during 2003–2006, is unexpected and likely represent gross under-reporting, considering the heavy industrial activity in Chukotka, which annually produces 20–25 tons of pure gold alone, as well as other metals and coal.

## International comparison of occupational diseases rates

According to the Organisation for Economic Co-operation and Development (OECD), the rate of ODs in Russia is considerably lower (some 10–100 times lower) than other circumpolar countries (16).

World health Organisation data for 2005 (Table I) show that Russia, countries who are formerly part of the USSR and former socialist countries in Eastern Europe all reported extremely low rate of ODs compared to Scandinavian countries (17).

**Table I:** Rate of new cases of occupational diseases (OD) in selected European countries, 2005

Country	Rate of OD (per 10,000 employed)
Former USSR	
Russian Federation	1.7
Ukraine	1.8
Belarus	0.3
Estonia	1.1
Lithuania	5.9
Latvia	16.2
Former socialist countries, Eastern Europe	
Czech Republic	1.8
Croatia	0.4
Slovakia	1.1
Hungary	0.7
Nordic countries	
Denmark	39.7
Norway	7.5
Sweden	34.9
Finland	19.4

Source: World Health Organisation (cited in 17).

Latvia is unique among former USSR countries in having a high level of ODs. Latvia instituted reforms after independence (17), with legislation regulating relations between employers and employees in the field of social insurance, obligatory medical examination of workers (paid for by the employers), improvement in the training and certification of occupational health specialists, the creation of state ODs register, and so on. Quantity of the certified professionals increased in Latvia by 8.4 times between 1996 and 2007. The number of ODs in Latvia increased 9 times during 1996–2004, from 20 to 185 cases per 100,000 workers. This increase is not the result of a worsening of workers’ health but the improvement of surveillance with more comprehensive coverage and accurate enumeration.

## History of occupational disease surveillance in USSR and Russia

The history of registration of ODs in the USSR/Russia is well described in the introduction of the doctoral dissertation by L.G. Zhavoronok from the Research Institute of Medicine of Labour, Russian Academy of Medical Sciences, Moscow (18).

The obligatory system of registration and reporting of ODs in Russia began in 1924 by the joint Decision of the National Commissariats of Work and of Public Health Services *About the obligatory notice on occupational poisonings and diseases*. Before that time, there were separate registrations for certain groups of occupational illnesses, mainly, acute poisonings, occupational infections, caisson illness and various accidents.

In 1939, the National Commissariat of Public Health Services adopted a new *Principle of the notice and registration of occupational poisonings and occupational diseases*, which introduced a person-based system of patients with ODs. Changes were made to the reporting form over the years, but essentially the system established in 1939 existed until 1986, for almost 50 years. It did not contain data on the cause of occupational disease, adverse factors influencing the patient, character of performed work, actions directed at eliminating these factors or health status of the patient at the moment of disease investigation. Even basic data, such as sex, age, occupational route, work experience, and so on, were not captured. The system did not provide sufficient information to analyse causes and effects.

A new system of obligatory registration of ODs was introduced in the USSR in 1986 and in Russia in 1991. In Russia, the state sanitary-epidemiological service (Gossanepidnadzor, now Rospotrebnadzor) of the Ministry of Health Care of the Russian Federation provides the centralised gathering of primary materials for registration of ODs. An important innovation is the unified form of “Sanitary-hygienic characteristic of workplace conditions in case of assumption of occupational disease (poisoning)” and instructions on its completion. The sanitary-hygienic characteristic of workplace is made by Rospotrebnadzor centre, taking into account the preliminary diagnosis of occupational disease, characteristics of all harmful factors of the occupational environment, labour process and modes of work, which could lead to occupational disease (poisoning). This is the major document confirming, or denying, the occupational character of disease (18).

The bureaucratic system and procedural complexity in establishing preliminary and definitive diagnosis of an occupational disease often lead to frequent occurrence of conflict situations, delay of a thorough medical examination of a patient in a specialised centre of occupational health, possibility of concealment of diseases and judicial proceedings.

The Federal centre of Rospotrebnadzor carries out the analysis of ODs in Russia, compiles annual reports and publishes newsletters. However, the published information does not provide sufficient details for different regions in Russia, separate sectors of the economy and different occupations. Different types of ODs and poisonings are presented only as a proportion of all ODs. Methodological and statistical errors occur frequently in the bulletins. There is no interaction between Rospotrebnadzor centres and the treatment and prevention facilities in which the diagnosis of an occupational disease is first established and patient supervision carried out. Reform of the current situation is badly needed (18).

## Review of published literature in Russian

Russian hygienic scientific journals have not yet embraced the electronic age. For example, *Gig Sanit* (Hygiene and sanitation) published since 1922 has a web page only with a list of the names of articles from 1998 (abstracts since 2010), while *Med Tr Prom Ekol* (Medicine of Labour and Industrial Ecology) published since 1957 has a much shorter list – since 2008; both journals are indexed in Index Medicus and in many other web-based bibliographic systems, but full text articles are unavailable. Russian special medical web searching systems are only at the early stages of development; comprehensive thematic catalogues do not exist. PubMed does contain names of articles from almost all major Russian biomedical and public health journals since the end of 1960s, and thus could be an important resource for searching Russian language articles.

The libraries inside Russian research institutes have faced serious financial problems since 1990. Today, across the vast country, only the Russian National Library in St. Petersburg, Russian State Library in Moscow and several other libraries in the biggest cities remain the repositories of comprehensive Russian scientific literature. Stocks of these libraries are electronically catalogued only partially, and most full text articles are available only in hard copies.

A systematic search of the Russian peer-reviewed literature on occupational health and disease was conducted for the period 1980–2010 using PubMed and electronic catalogues of the Russian National Library in St. Petersburg. The specific terms “Arctic” and “North” were not initially included to search as broadly as possible. Publications specific to the Russian Arctic, Siberia and Far East were then further identified.

Most of the articles on occupational health are descriptive in nature, assessing single exposures (such as air pollutants, noise, vibration, temperature, etc.); others deal with physiological, cardiac, immune and neuro-hormonal status in occupational conditions but without analysis of exposure–effect association; others present data on time lost at work due to general sickness (such as colds and diarrhoea, etc.) at industrial enterprises. The review below is focused on critical evaluation of the occupational health care system in Russia.

Initial timid attempts at criticism of the quality of medical examination of workers began to appear in Russian scientific journals in 1986, soon after Gorbachev's coming to power and the beginning of “perestroika”.

In 1982–1984, during the construction of gas pipelines from Urengoy (in the Yamal peninsula), there was on average one doctor for 328 workers engaged in dangerous and hazardous job conditions, and 83 patients from the general population. A mobile medical team moved with the workers as the construction progressed. A questionnaire survey in Urengoy showed that 11% of workers complained about difficulties in getting to see the doctor. Some 74% of workers considered themselves to be overexposed to noise and vibration and 78% to petrochemicals. Housing conditions were generally poor, with 81% living in wagons or “dog houses” (4–5 m^2^/person). There were serious lifestyle issues, with 79% smokers, and 77% consumed on average 250–500 ml of vodka in a single dose (19).

By the end of the 1990s, more critical articles on the system of diagnosis and registration of ODs appeared in Russian scientific journals. In 1997, the Research Institute of Hygiene and Occupational Pathology in Nizhniy Novgorod noted the substantial increase of ODs in Nizhniy Novgorod region since 1990, which the author attributed to more frequent visits of workers to doctors due to the worsening of the economic situation and the wild market economy, with 80% of enterprises in the region privatised (20). There was an increase in chronic OD rate, while the number of disabled workers and general degradation of the OD detection system were discussed in connection with dramatic changes towards the wild market economy. The author identified an urgent need of holding employers legally accountable for neglecting job safety rules and for under-reporting ODs (20).

In 1998 the nickel-cobalt industry in the Russian Arctic was the subject of an assessment by the Kola Research Laboratory of Occupational Health in Kirovsk city, Murmansk Oblast (21). Despite considerable financial inputs, substantial improvement of working conditions had not occurred. Occupational disease level among nickel-cobalt industry workers was very high, but it was estimated that only 2% of ODs were detected, based on detailed medical examination of the workers, with the true prevalence likely around 510 cases per 10,000 workers (21).

By the end of the 2000s, numerous articles disclosing and unmasking the Russian system of occupational health care and OD registration have appeared on the pages of the most respectable journals. Among the vocal critics was Gennady Onishchenko, the Chief of Sanitary-Epidemiological Surveillance (Chief State Sanitary Officer) of the Ministry of Health Care and Social Development, academician of the Russian Academy of Medical Sciences (22). Some of the main points of his article in the journal *Hygiene and Sanitation* are highlighted below.

In 2006, 23% of workers employed in main Russian industries worked in job conditions that violated sanitary-hygienic rules. Almost 80% of all Russian industrial enterprises were categorised as dangerous or hazardous for health. The worst labour conditions are found in coal mining, ship building, ferrous and non-ferrous metallurgy, agriculture, tractor and agricultural machinery construction, building materials production, lumbering and construction activities. The highest number of people who work in dangerous and hazardous conditions are employed at non-governmental enterprises. In many regions of Russia, the budget for improving labour conditions has been cut dramatically. In many enterprises, their hygienic laboratories were eliminated or their financing was reduced significantly (22).

Industrial recession and economic instability have affected many enterprises, resulting in dilapidation and disrepair of buildings, machines and equipment. Much of the Russian industry still relies on manual labour (accounting for 70% of production), with a low level of mechanisation and automation. Many companies blatantly ignore or violate occupational safety regulations relating to noise, vibration, temperature, air quality, illumination, personal protective gear, duration and schedule of work, limits for lifting and carrying of weights, and so on.

Industrial reorganisation involving partition and mergers of industrial resources and properties has resulted in the emergence of new legal entities with very limited or no responsibility for the health and safety of workers. Some of the new enterprises are located on temporary rented sites where employers do not want to invest in infrastructure improvement.

In Russia, official statistics show a continuing decline in the rate of ODs in the face of worsening labour conditions, a paradox that can be explained by inadequate monitoring and reporting. Among the factors responsible for this state of affairs are:

- no coverage and low quality of preventive health examination of workers;
- employers’ disinterest in detecting ODs to maintain low insurance premiums and avoid costs associated with improving labour conditions and health care measures;
- lacklustre performance of medical examiners, particularly when the employer has a long-term arrangement with patient care institutions;
- workers’ tendency to hide early symptoms of disease, which will affect their ability to continue employment; on the other hand, later detection after the onset of disability will bring in significant compensation for the family; and
- inadequate labour protection legislation.

Additional criticism of the problem was expressed by other specialists from the Research Institute of Labour Medicine in Moscow. The number of disabled workers in Russia increased dramatically from 7.9 million in 1997 to 13 million in 2007, when disabled workers constituted 11% of the adult population of Russia (23). The fears of unemployment among workers (a new phenomenon in Russia), the low regard for human life (an old phenomenon in Russia), and the quest for profits by employers jointly produced a situation of widespread worsening of job conditions, and the physical and psychological exhaustion among workers.

Official statistics of Magadan Oblast show incredibly low values of OD (1.3–3.2 cases per 10,000 workers) in 2004–2008 (14). The Angarsk Research Institute of Labour Medicine and Human Ecology evaluated the registration of OD among miners in Magadan Oblast between 1981 and 2000 and found that the real situation is very different from official statistics. For the mining industry as a whole in Magadan Oblast, OD rate among all workers fluctuated between 35 and 61 cases per 10,000 workers, 52 and 93 cases per 10,000 workers among above-ground workers and 266 and 398 cases per 10,000 workers among miners working underground. For specific conditions such as vibration disease, among underground miners who had been exposed to vibration for 5 or more years, 64% had signs of vibration disease. Vibration tends to develop about twice as fast in the Arctic than among workers in middle latitudes, a contributing factor being the below freezing temperatures often found underground (24).

An assessment of Krasnoyarsk region by the regional centre of occupational pathology and Krasnoyarsk Medical University (25) found that in the 1990s, much of previous gains in occupational health care had been lost – the number of medical units at enterprises was considerably reduced, preventive work decreased, and the quality of medical examinations and diagnosis of ODs declined. In Krasnoyarsk region, every second worker works in conditions below hygienic standards. With the intensification of medical examination of workers, the proportion of workers screened by surveys increased from 80% in 1998 to 91% in 2008 (25).

Between 1978 and 1988, official OD levels in Archangelsk city were comparable to those in Archangelsk Oblast and Russia as a whole (0.8–1.8 per 10,000 of workers), but during the next decade (1989–2000), the rate for Archangelsk city increased dramatically to 14.1/10,000, which was more than 7 times higher than in Russia. This is the result of the launching of the department of occupational pathology in Archangelsk city and expert assessment of the quality of medical examination of workers. However, in 2000–2002, the OD level in Archangelsk city again fell to the level of the 1980s due to the cessation of expert work (26).

## Conclusions

This review presents official statistical data on occupational disease and summarises Russian published peer-reviewed scientific literature on occupational health care in Russia and the Russian Arctic in 1980–2010.

If Russian official statistics are to be trusted, the country and its northern regions have extremely low rates of ODs, substantially lower (some 10–100 times) than other circumpolar countries with advanced social welfare systems and high occupational health and safety standards. This positive picture is at odds with the poor compliance with occupational health regulations, and the breakdown in occupational health monitoring and workers’ health programmes, which were exacerbated by the “wild market” economy since the 1990s and accompanying major industrial restructuring in the North and elsewhere.

Reform is urgently needed across Russia. Legislation is needed to hold employers accountable for adherence to safety regulations and truthful reporting of ODs and injuries. Where monitoring and surveillance systems have been put in place, there has been a corresponding increase in reported ODs. Well-equipped and staffed hygienic laboratories and regular health screening of workers by qualified personnel in both state and private enterprises require adequate financial and administrative support. There is some evidence that the very poor state of occupational health and health care is receiving high-level governmental attention. What is needed is implementation of sound principles and policies.
